# Resting costs too: the relative importance of active and resting energy expenditure in a sub-arctic seabird

**DOI:** 10.1242/jeb.243548

**Published:** 2022-02-16

**Authors:** Fred Tremblay, Shannon Whelan, Emily S. Choy, Scott A. Hatch, Kyle H. Elliott

**Affiliations:** 1 Department of Natural Resource Sciences, McGill University, Ste Anne de Bellevue, QC, Canada, H9X 3V9; 2 Institute for Seabird Research and Conservation, Anchorage, AK 99516, USA

**Keywords:** Energetics, Physiology, *Rissa tridactyla*, Thyroid, Biologging, Accelerometry

## Abstract

Breeding is costly for many animals, including birds that must deliver food to a central place (i.e. nest). Measuring energy expenditure throughout the breeding season can provide valuable insight into physiological limitations by highlighting periods of high demand, and ultimately allows improvement of conservation strategies. However, quantifying energy expenditure in wildlife can be challenging, as existing methods do not measure both active (e.g. foraging) and resting energy costs across short and long time scales. Here, we developed a novel method for comparing active and resting costs in 66 pre-breeding and breeding seabirds (black-legged kittiwakes, *Rissa tridactyla*) by combining accelerometry and triiodothyronine (T3) as proxies for active and resting costs, respectively. Active energy costs were higher during incubation (*P*=0.0004) and chick rearing (*P*<0.0001) than during pre-laying, because of an increase in the time spent in flight of 11% (*P*=0.0005) and 15% (*P*<0.0001), respectively. Levels of T3, reflecting resting costs, peaked marginally during incubation with a mean (±s.d.) concentration of 4.71±1.97 pg ml^−1^ in comparison to 2.66±1.30 pg ml^−1^ during pre-laying (*P*=0.05) and 3.16±2.85 pg ml^−1^ during chick rearing (*P*=0.11). Thus, although chick rearing is often assumed to be the costliest breeding stage by multiple studies, our results suggest that incubation could be more costly as a result of high resting costs. We highlight the importance of accounting for both active and resting costs when assessing energy expenditure.

## Abstract (precis)

**Summary:** Measurements of both active and resting energy expenditure in breeding black-legged kittiwakes suggest that chick rearing may not be the costliest breeding stage, highlighting the need to measure both active and resting cost when assessing energy expenditure.

## INTRODUCTION

Energy is a fundamental currency in ecology, and understanding metabolic constraints on wildlife has enabled us to implement more effective conservation strategies (Wikelski and Cooke, 2006). An individual's energy expenditure is driven by environmental (e.g. temperature) and internal factors (e.g. breeding stage). Breeding is a costly event for many animals, especially birds that must deliver food to a central place (i.e. the nest; Elliott et al., 2009). During breeding, individuals balance investment in their offspring against investment in their own subsequent survival (Sibly and Calow, 1986; Williams, 1966; Welcker et al., 2015). If parents cannot balance these investments and external requirements are higher than the energy resources available to them, individuals reach physiological overload (Wikelski and Cooke, 2006). Once such overload is reached, individuals have to physiologically and behaviourally adapt to survive. If the overload persists over time, a decrease in breeding success and correspondingly in population size can follow (Wikelski and Cooke, 2006). Identifying the most energetically demanding stages can inform us about the period during which physiological overload is reached. Hence, measuring energy expenditure over the length of the breeding season can provide valuable insight into physiological limitations.

In order to cope with periods of high physiological demands (e.g. breeding), resting costs can be downregulated to allocate more energy toward active energy expenditure. This is known as the ‘compensation hypothesis’, where active and resting costs are regulated independently. Such a strategy prevents individuals from reaching allostatic overload when their energy expenditure becomes too high. Alternatively, the ‘potentiation hypothesis’ states that active cost changes are matched by a corresponding change in resting cost. Using clutch manipulation and supplemental feeding, Welcker et al. (2015) demonstrated that the compensation hypothesis is supported in a seabird species. Additionally, Whelan et al. (2021) demonstrated that supplemental feeding did not affect energy reserves, but rather impacted breeding phenology.

To obtain estimates of energy expenditure that reflect the total cost of breeding, both resting and active (e.g. foraging) costs must be measured. Resting costs can be assessed by measurement of the thyroid hormone triiodothyronine (hereafter T3; Hollenberg, 2008; McNabb, 2007; Brinkmann et al., 2016). T3 is well known for its role in tissue oxygen consumption and thermogenesis and is positively associated with resting metabolic rate in many homeotherms (warm-blooded animals; Hollenberg, 2008; Elliott et al., 2013; Welcker et al., 2013). Active costs can be measured using accelerometry. Once calibrated, accelerometers enable measurement of activity-specific metabolic rate based on dynamic body acceleration (Gabrielsen et al., 1987; Jodice et al., 2003; Stothart et al., 2016). Accelerometers are also frequently used to monitor wildlife and obtain detailed information about animal movements and behaviour over time (time–activity budgets) from which we can infer active energy expenditure (Ropert-Coudert et al., 2009; Brisson-Curadeau et al., 2017). Accelerometry offers an easy way to study energy expenditure in challenging species, such as seabirds, which are highly pelagic, spending most of their time at sea (Cook et al., 2017; Hicks et al., 2018).

The black-legged kittiwake, *Rissa tridactyla* (Linnaeus 1758) (hereafter kittiwake), a species of cliff-nesting gull, has been studied extensively, yet studies looking at their energetic costs throughout the breeding season are still lacking. Chick rearing has been suggested as the costliest breeding stage because kittiwakes forage more intensively to provide for their chick and, hence, spend more time flying (Golet et al., 1998). The high cost of flying has been highlighted in kittiwakes (Gabrielsen et al., 1987). Studies focused solely on resting costs showed that kittiwakes increased their resting metabolic rate during incubation, which then decreased during chick rearing (Langseth et al., 2000; Welcker et al., 2013). While most studies measure daily energy expenditure during incubation and chick rearing (Langseth et al., 2000; Bech et al., 1999; Moe et al., 2002; Gabrielsen et al., 1987; Jodice et al., 2002), little is known about the energy expenditure of pre-laying kittiwakes. Measurements of active and resting energetic costs throughout the breeding season (i.e. pre-laying, incubation, chick rearing) are still needed to better understand the physiological constraints that kittiwakes experience during this period of high energy demand.

Here, we further developed a method for comparing active and resting costs in kittiwakes using accelerometry (active costs) and T3 (resting costs). Our approach builds on that of Welcker et al. (2015), who used doubly labelled water (DLW) to measure total costs and T3 to measure resting costs but could not directly measure active costs. We measured the time–activity budgets of adult kittiwakes during pre-laying, incubation and chick-rearing stages. Using a DLW–movement calibration published by Jodice et al. (2003), we estimated daily energy expenditure (DEE) based on time–activity budgets, allowing us to obtain fine-scale estimates of active costs, independently from variations in resting metabolic rate. We also examined how free T3 concentration (unbound T3 circulating in the blood; Welcker et al., 2015) fluctuates over a whole breeding season in individual adults and compared variation in resting costs with that in active costs. We hypothesized that if chick rearing is the most demanding phase for breeding kittiwakes, then time spent flying and activity-related DEE will be higher during chick rearing. Additionally, we hypothesized that if kittiwakes experience higher resting costs during incubation because of the need to warm the eggs, then free T3 levels will be higher during incubation, as also observed by Welcker et al. (2013). Finally, we hypothesized that individual variation in pre-laying DEE will be greater in females than in males, as females tend to reduce their foraging activity as they approach their egg-laying date.

## MATERIALS AND METHODS

### Experimental design

We studied kittiwakes breeding on Middleton Island, Gulf of Alaska (58″25′N, 146″19′W), from 19 May to 27 July 2019. Kittiwakes were nesting on an abandoned radar tower that had been adapted for research purposes. Each nest was equipped with a one-way mirrored window, which allowed us to monitor each breeding pair while reducing disturbance. We identified individuals in the field based on their unique combination of coloured Darvic bands and sexed them based on behaviour. To assess the time–activity budget of kittiwakes, we deployed GPS-accelerometers on kittiwakes (*n*=72) spread across breeding stages (20 pre-laying, 20 incubation, 32 chick rearing) and sexes (34 females, 38 males). To assess the active DEE of kittiwakes, we coupled these time–activity budgets with the activity-specific metabolic rates of kittiwakes published by Jodice et al. (2003). All work was approved by the McGill Animal Care Committee (protocol #2016-7814), under state permit 19-137 issued by the Alaska Department of Fish and Game and federal permit 85004-C issued by the US Fish and Wildlife Services.

We deployed GPS-accelerometers (8 g, AxyTrek, Technosmart) on all birds (*n*=72) for 2 days. The devices recorded GPS location every 3 min and tri-axial acceleration at 25 Hz. We placed the units on the two central rectrices, including some coverts, approximately 1.0 cm below the uropygial gland. We fixed the units using superglue, marine cloth tape (Tesa tape) and cable ties. We deployed GPS-accelerometers on pre-laying birds that had nests ranging from non-existent to fully developed, on incubating birds that had eggs older than 15 days, and on chick-rearing birds with chicks between 5 and 15 days. Based on those criteria, we deployed devices opportunistically every 3–8 days from mid-May to late July.

Upon deployment and retrieval of GPS units, we collected 1 ml of blood from the brachial vein of kittiwakes within 3 min of capture (using a 25G needle and a heparinized syringe) to obtain a total of two blood samples per bird in 2 days (*n*=68). Upon collection of the samples, we spun down the whole blood and collected plasma which we then immediately froze at −20°C. Samples remained frozen for the rest of the field season (1–3 months) and during the transport back to McGill University (using dry ice) and were stored in a −20°C freezer until analysed for free T3 levels.

### Utilization distribution

We used GPS locations to calculate the residence in time and space (hereafter referred to as ‘utilization distribution’) and assessed foraging location, foraging distance and overall distribution across the landscape of kittiwakes. Foraging location was defined as areas where kittiwakes conducted area-restricted search (Torres et al., 2017). We calculated utilization distributions of foraging locations (50%, 85%, 95%) with *adehabitatHR* (Calenge, 2006). We then compared utilization distributions across the breeding stages to account for potential differences in foraging distance and/or location, which could affect DEE and time–activity budgets of breeding kittiwakes.

### Time–activity budgets

To obtain time–activity budgets, we processed accelerometer data to calculate wingbeat frequency (methods in Patterson et al., 2019) and GPS data to assess the distance from the colony. We used wingbeat frequency and distance from the colony (near: <500 m from colony; far: ≥500 m from colony) to classify behaviour as in flight, at the colony or on water using Hidden Markov Models (*momentuHMM*; McClintock and Michelot, 2018). For each kittiwake, we calculated the proportion of time spent at the colony, on water and in flight.

### DEE

To assess active energy expenditure, we estimated DEE using the accelerometry calibration published by Jodice et al. (2003) based on the formula published in Stothart et al. (2016):
(1)

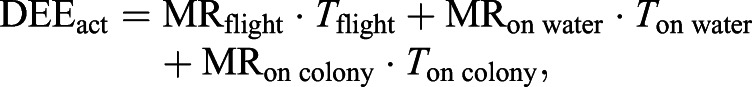
where DEE_act_ corresponds to daily energy expenditure, MR corresponds to the activity-specific metabolic rate of birds in a given behaviour (flying, swimming or at the colony), and *T* corresponds to the proportion of time spent in that behaviour (percentage of the day). We used activity-specific metabolic rates that correspond to our behavioural classification from similar behavioural classes used by Jodice et al. (2003) to create their calibration (Table 1). To convert their energy estimates from ml CO_2_ to J, we used an average caloric equivalent from a multi-year study on kittiwakes (27.63 J ml^−1^ of CO_2_; Welcker et al., 2010). Using the activity-specific metabolic rates, we obtained an estimate of DEE, based on the activity component of energy expenditure.

**Table 1.**
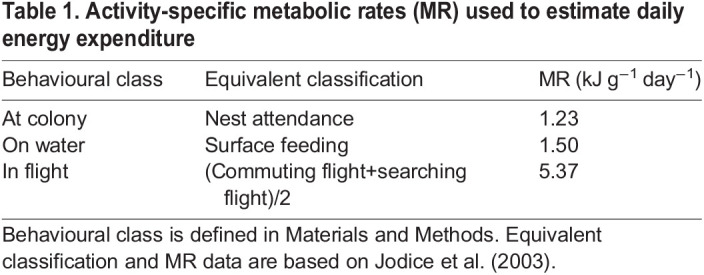
Activity-specific metabolic rates (MR) used to estimate daily energy expenditure

### T3 hormone

We used free T3 levels, a proxy for resting metabolic rate, to assess the resting component of DEE. We conducted an enzyme immunoassay (ELISA) to quantitatively assess the concentration of free T3 in the kittiwakes’ blood (MP Biomedicals catalogue number 07BC-1006, Irvine, CA,USA). Each blood sample was run in duplicate and a standard curve was obtained for each run to ensure the replicability between assays. For birds that were sampled twice, we averaged free T3 concentration of both initial and final blood samples.

### Statistical analysis

We modelled changes in body mass (body condition) from deployment to final retrieval (linear model) in response to duration of deployment (in hours) and sex. We conducted an analysis of variance (ANOVA) on the model to test for both interactive (ANOVA type III) and main effects (ANOVA type II) of deployment duration and sex on body condition. To test for impacts of tagging on body condition, we conducted a paired *t*-test to assess whether mass had decreased significantly during the length of the deployment for both males and females.

We used linear models to test for effects of sex and breeding stage on time–activity budget, DEE and T3. For each model, we conducted an ANOVA to test for interactive (ANOVA type III) and main effects (ANOVA type II). If the ANOVA test revealed significant effects of sex and/or breeding stage, we conducted an analysis of least-squares means (LSM). All analyses were run using R. 3.6.3 (http://www.R-project.org/) and the *emmeans* package (https://CRAN.R-project.org/package=emmeans) and significance was judged at α=0.05.We made all figures using *ggplot2* (Wickham, 2016; https://ggplot2.tidyverse.org) and *leaflet* (http://cran.r-project.org/package=leafletR)*.* Data are reported as means±s.d.

## RESULTS

### Experimental design

We found no interactive or main effects of deployment duration and sex on the birds’ body condition (Table 2). Out of 72 birds equipped with GPS-accelerometers, we obtained usable data for 66 individuals. For the remaining six birds, four were not recaptured, one lost the device and one device was damaged. A two-tailed paired *t*-test showed that body mass (*M*) did not decline significantly over the course of the deployment in both females (*M*_pre_=392±7 g, *M*_post_=385±7 g; paired *t*_30_=1.70, *P*=0.09829) and males (*M*_pre_=425±7.7 g, *M*_post_=419.43±5.97 g; paired *t*_34_=1.04, *P*=0.3045). On average, deployments lasted 2.42±1 days (range: 1.77–2.98 days).

**Table 2.**
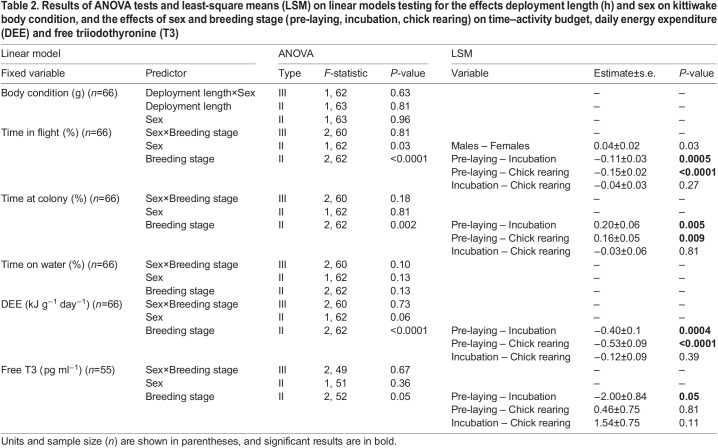
Results of ANOVA tests and least-square means (LSM) on linear models testing for the effects deployment length (h) and sex on kittiwake body condition, and the effects of sex and breeding stage (pre-laying, incubation, chick rearing) on time–activity budget, daily energy expenditure (DEE) and free triiodothyronine (T3)

### Utilization distribution and time–activity budget

**Fig. 1.**
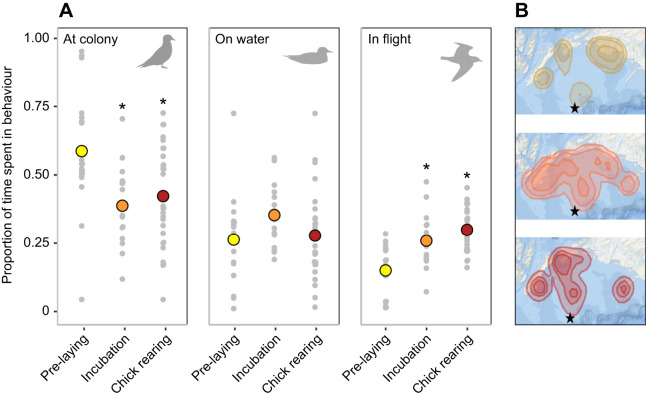
**Time–activity budget and utilization distribution of black-legged kittiwakes (*n*=66) across three breeding stages.** (A) Proportion of time spent at the colony, on the water and in flight. Significant difference from pre-laying is indicated by an asterisk. (B) Utilization distribution (from top to bottom): 95%, 75% and 50%. Colony location is indicated by a black star.

Birds foraged at a similar distance from the colony, but shifted their time–activity budgets throughout the breeding season (Fig. 1). Kittiwakes significantly increased the time spent in flight after pre-laying, by 11±3% during incubation and 15±2% during chick rearing (Table 2). Males spent overall slightly more time in flight than females (Table 2). Kittiwakes also decreased their time spent at the colony after pre-laying by 19±6% during incubation and 16±5% during chick rearing (Table 2). Males and females showed no significant difference in time spent at the colony across the breeding season (Table 2). Time spent on water did not vary across the breeding season or with sex (Table 2).

### DEE

**Fig. 2.**
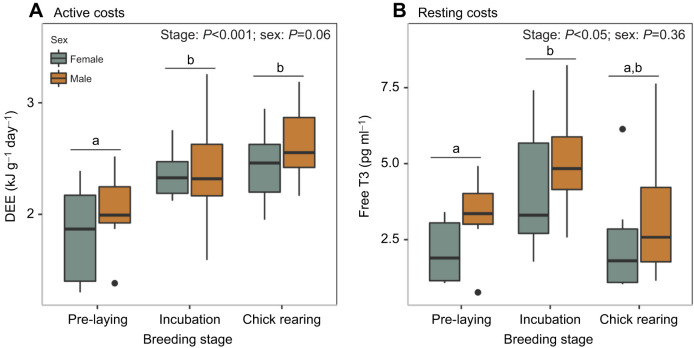
**Daily energy expenditure (DEE) and free triiodothyronine (T3) levels in black-legged kittiwakes across three breeding stages.** Effects of sex and breeding stage on (A) active costs based on time–activity budgets (*n*=35 male; *n*=31 female) and (B) resting costs using free T3 concentration (*n*=28 male; *n*=27 female). Different letters indicate a significant difference between breeding stages.

Kittiwakes had a DEE of 1.64±0.32 kJ g^−1^ day^−1^ during pre-laying, 2.05±0.34 kJ g^−1^ day^−1^ during incubation and 2.17±0.27 kJ g^−1^ day^−1^ during chick rearing (Fig. 2). After accounting for mass differences, males and females spent an equivalent amount of energy throughout the breeding season (Table 2). Kittiwakes significantly increased their DEE during incubation and chick rearing relative to pre-laying (Table 2).

### T3 hormone

Based on samples obtained from 27 females and 28 males, we observed a notable increase in free T3 concentration during incubation. Free T3 peaked during incubation with an average concentration of 4.71±1.97 pg ml^−1^, against 2.66±1.30 pg ml^−1^ in pre-laying and 3.16±2.85 pg ml^−1^ in chick rearing (Fig. 2). Kittiwakes’ free T3 level increased significantly between pre-laying and incubation whereas the variation in free T3 between pre-laying and chick rearing and incubation and chick rearing was non-significant (Table 2). Free T3 concentration did not vary according to sex throughout the breeding season (Table 2). These results show that resting energy expenditure increases significantly during incubation compared with pre-laying, and that chick-rearing birds have similar resting energy expenditure to that of both pre-laying and incubating birds.

## DISCUSSION

Kittiwakes increased their active costs during incubation and chick rearing relative to pre-laying; resting costs were also higher during incubation compared with pre-laying, but chick-rearing levels were not significantly different from either pre-laying or incubation levels (Fig. 2). The increase in active costs in incubation and chick rearing was driven by an increase in time spent in flight. Males and females exhibited similar time–activity budgets (Table 2) and, consequently, similar activity-driven DEE. Energy expenditure was more variable among females during pre-laying. The concentration of T3 increased during incubation compared with pre-laying but was similar to chick-rearing levels, which suggests a similar trend for the resting metabolic rate of breeding kittiwakes (Fig. 2).

Unlike what we first hypothesized, chick rearing was not the most demanding phase based on active costs, as DEE during incubation and chick rearing was similar. Active costs were mainly driven by the increase in time spent flying during both breeding stages. However, as we used a single metabolic rate estimate for flight, we did not account for changes in respiratory exchange ratio based on the time kittiwakes spent in flight, a potential source of error in our DEE estimates (Rothe et al., 1987). As foraging distance was similar across all breeding stages (Fig. 1), this is most likely a response to more numerous foraging trips (Osborne et al., 2020). An increase in foraging effort during chick rearing has been described in many seabird species, as parents must provision their chicks (Golet et al., 1998). However, the high number of foraging trips during incubation suggests that parents themselves experience higher energetic needs, potentially as a response to high thermoregulatory costs (Norton, 1973).

The concentration of T3 was highest in incubation as predicted by our second hypothesis; however, there was no significant difference between T3 concentrations in incubation and chick rearing. The increased DEE and levels of T3 hormones during incubation could both suggest high thermoregulatory costs (i.e. costs of keeping their eggs warm). Although incubation and chick rearing were statistically similar, our results are consistent with other studies comparing the energy expenditure and resting metabolic rates of incubating and chick-rearing kittiwakes where T3 peaked quickly in incubation and slowly decreased in chick rearing (Thomson et al., 1998; Elliott et al., 2013; Welcker et al., 2013; Criscuolo et al., 2003). High T3 levels are associated with high body temperatures (Welcker et al., 2013), such as when parents use their body temperature to warm their eggs. However, low T3 during chick rearing may be the result of a trade-off, where adults lower their resting metabolic rate to save energy during this costly breeding stage (Welcker et al., 2013). As our T3 analysis was conducted using an ELISA kit, a direct comparison of energy equivalents was not possible because previous studies analysed T3 using radioimmunoassay (Welcker et al., 2013; Elliott et al., 2013). However, a previous study showed that basal metabolic rate (BMR) costs accounted for approximately 33% of daily energy expenditure (average field metabolic rate, FMR=22.3±0.1 W kg^−1^ and average BMR=7.4±0.3 W kg^−1^) in early chick-rearing kittiwakes (Fyhn et al., 2001). Although the direct cost of thermoregulation in incubating kittiwakes has not been investigated yet, it has been studied in other bird species (Gabrielsen et al., 1991), such as shorebirds with biparental care, where embryos near hatching contributed to 35–40% of the cost of incubation in both parents (Norton, 1973). Alternatively, high T3 levels might be the result of increased heat loss from the brood patch (Tapper et al., 2020), especially when spending time on water (Umeyama et al., 2021). However, heat loss to the environment was adequately mitigated via vasoconstriction in the brood patch of black grouse (a subarctic species) when not incubating (Tøien, 1993).

We observed high individual variation in time–activity budgets throughout the breeding season (Fig. 1). We believe that this individual variation can be attributed in part to our short sampling interval (∼2 days limited by DLW), as some individuals did not forage during the deployment whereas others spent most of the deployment foraging while their partner attended the nest. Changing conditions between deployments likely contributed to high individual variation as kittiwakes are known to adapt their foraging strategies, with foraging trips ranging from a few hours to over a day (Kotzerka et al., 2010). Additional variation in pre-laying could also be attributed to females that were at a variable number of days from laying date. For example, some females laid the day following unit retrieval, while others laid several days later. Individual variation in time–activity budgets (Fig. 1) and DEE (Fig. 2A) of pre-laying females could be driven by reduced activity when they approach laying; as the egg develops and females get heavier, they reduce time spent foraging and spend more time at the colony (Whelan et al., 2021; Creelman and Storey, 1991). For future studies, we believe that increasing the sampling interval and using only males could reduce individual variation in time–activity budgets and DEE. However, overall, we believe that our sample size was sufficiently large to detect general trends in behaviour that reflect the activity of kittiwakes across the breeding season on Middleton Island.

Males and females had similar energy expenditure after accounting for total body mass. Although we did not control for environmental conditions, females and males were deployed simultaneously regardless of weather from June to August, meaning that they met an array of conditions. We are confident that the long period over which we deployed birds acted as an appropriate control for environmental conditions and they did not affect our results significantly. Based on the variation observed in pre-laying and the lack of difference detected between males and females, we suggest that males might be more suitable for further calibration experiments.

Our study is one of the first to investigate both active and resting costs throughout a breeding season. Chick rearing has been identified as the costliest breeding stage by multiple studies (Golet et al., 1998; Moe et al., 2002; Bech et al., 2002). Our results suggest that incubation could be more costly than previously thought, when accounting for both active and resting energy expenditure. As temperatures are likely to increase with climate change (Esch and Osterkamp, 1990; Royer and Grosch, 2006), thermal stress on an individual's metabolism is likely to affect them at a much faster rate than indirect effects (e.g. phenological mismatch, novel species interaction, range shifts, etc.), and pose challenges to avian thermoregulation and endocrinology (Ruuskanen et al., 2021). Increased temperature might also result in lower resting costs in incubation by reducing the need for heat production. Hence, both resting and active costs should be considered when measuring energy expenditure. As the current calibration published by Jodice et al. (2003) only accounts for active costs in chick-rearing birds, a more robust calibration is needed to accurately assess energy expenditure prior to and during the breeding season. We suggest that resting costs should be incorporated into any further calibration experiment.
